# Clinical experience in open robotic-assisted microsurgery: user consensus of the European Federation of Societies for Microsurgery

**DOI:** 10.1007/s11701-025-02338-w

**Published:** 2025-04-22

**Authors:** Maximilian Kueckelhaus, Alexandru Nistor, Tom van Mulken, Emre Gazyakan, Khaled Dastagir, Henning Wieker, Maria Mani, Shan Shan Qiu, Jens Ahm Sørensen, Gemma Pons, Peter Vogt, Jaume Masia, Ulrich Kneser, Pierluigi Tos, Sinikka Suominen, Marco Innocenti, Nicole Lindenblatt

**Affiliations:** 1 Department of Plastic Surgery, University Hospital Muenster, University of Muenster, Albert-Schweitzer-Campus 1, 48149 Muenster, Germany; 2 Department of Plastic Surgery, University Hospital Brussels, Brussels, Belgium; 3 Department of Plastic, Reconstructive and Hand Surgery, Maastricht University Medical Center, Maastricht, Netherlands; 4 Department of Hand, Plastic and Reconstructive Surgery, Burn Center, BG Trauma Center Ludwigshafen, Ludwigshafen, Germany; 5 Department of Plastic, Aesthetic, Hand and Reconstructive Surgery, Hannover Medical School, Hannover, Germany; 6 Department of Oral and Maxillofacial Surgery, Christian Albrechts University, UKSH Campus Kiel, Kiel, Germany; 7 Department of Plastic and Maxillofacial Surgery, Uppsala University Hospital, Uppsala, Sweden; 8 Department of Surgical Science, Uppsala University, Uppsala, Sweden; 9 Department of Plastic Surgery, Odense University Hospital, Odense, Denmark; 10 Plastic Surgery, Hospital de la Santa Creu i Sant Pau, Barcelona, Spain; 11 Department of Hand Surgery and Reconstructive Microsurgery, ASST Gaetano Pini-CTO, Milan, Italy; 12 Department of Plastic Surgery, Helsinki University and Helsinki University Hospital, Helsinki, Finland; 13 Department of Plastic Surgery, Rizzoli Orthopaedic Institute, Bologna, Italy; 14 Department of Plastic Surgery and Hand Surgery, University Hospital Zurich, University of Zurich, Zurich, Switzerland

**Keywords:** Robotic microsurgery, Robotic surgery, Microsurgery, Supermicrosurgery, Lymphatic surgery, Robotics

## Abstract

This study provides a comprehensive guide to robotic-assisted microsurgery. Following more than 900 clinical cases in 13 centers, this joint project was initiated to summarize and consent to the user experience. Two robotic systems specifically designed for the needs of open microsurgery received the first certification for clinical application (CE mark) in 2019 and 2020. Since their introduction into clinical application, several European microsurgical centers have implemented these systems, generating user experiences in multiple microsurgical subspecialties. All institutions using the MUSA-2 microsurgical robot (Microsure B.V., Eindhoven, Netherlands) or Symani Surgical System (Medical Microinstruments, Inc., Wilmington, Delaware, US) were invited to join the multicenter project. A modified nominal group technique was applied to answer five major questions regarding current and future indications and developments in open robotic-assisted microsurgery. Steep preclinical and clinical learning curves were characterized. General considerations concerning the two different systems are presented. Specifics for each microsurgical subspecialty are reported. Following two voting rounds, a consensus was reached in three of the five major questions with “lymphatics” being the top indication, “higher precision” being the top benefit, and “automation” being the top long-term goal of robotic-assisted microsurgery. This joint project of all Symani and MUSA-2 users presents clinical cases and the subsequent initial knowledge and experience. Lymphatics as a top indicator and a higher precision as the top benefit point toward the capabilities of robotic-assisted microsurgery in manipulation of smallest structures. Automation may further enhance and simplify robotic procedures in future. This project also provides a comprehensive guide to any institution aiming to introduce such a system for open robotic-assisted microsurgery in future.

**Supplementary Information:**

The online version contains supplementary material available at 10.1007/s11701-025-02338-w.

## Introduction

Since the initial clinical application of robotic assistance in complex surgical procedures in 1997[1], several surgical specialties have implemented robotic-assisted procedures into their clinical routine [2]. These systems were developed for minimally invasive surgery on macroscopic anatomic structures, and their application in microsurgery therefore was limited. Besides initial attempts of microsurgical anastomosis with the da Vinci system (Intuitive Surgical, Sunnyvale, CA, US) in 2007 [3], several groups have developed DIEP flap harvest approaches with the da Vinci [4–6]. The demands toward physical performance of microsurgeons are steadily increasing, for example with the introduction of perforator-to-perforator anastomosis [7, 8] and lymphatic supermicrosurgery [9, 10]. Consequently, the demand for technology supporting surgeons in surpassing their physical limitations increased.

In 2019 and 2020, two robotic systems developed methods to address the specific needs of open microsurgery, namely the MUSA-2 system (Microsure, Eindhoven, the Netherlands) and the Symani Surgical System (Medical Microinstruments, Inc., Wilmington, Delaware, the US) (Suppl. Fig. 1) and received approval for clinical application in Europe [11]. Being the first region worldwide to grant approval for clinical application, 13 European microsurgery centers implemented the MUSA-2 or the Symani in their practice. These centers performed over 900 clinical cases, marking the world’s initial experience in robotic-assisted free-flap surgery, lymphatic surgery, replantation, and more using these systems. Following standardized preclinical training and certification of every participating surgeon for clinical use, each of the 13 centers started individual clinical programs based on their distinct microsurgical spectrum and the areas of anticipated benefits. A joint project was initiated to summarize the initial clinical user experience with both systems and create a comprehensive guide for future institutions planning to implement robotic microsurgery.

## Methods

All institutions actively performing clinical robotic-assisted microsurgery by April 1 st 2024 with either the Symani Surgical System or the MUSA-2 System were contacted to participate in the multicenter project and attend to the consensus session at the European Federation of Societies for Microsurgery Meeting (EFSM) 2024 on May 9 th 2024. Twelve of the 13 active centers participated with at least one representative who was given the opportunity to present their individual experience (Suppl. Figure 2 and Suppl. Table 1).

### Modified nominal group technique (NGT)

A modified nominal group technique (NGT) was applied to answer five major questions regarding current and future indications and developments in open robotic-assisted microsurgery (method described in Suppl. Table 2).

The modified NGT was performed in four stages:

1. Silent generation.
2. Round robin.
3. Discussion
4. Voting.

Stage one of the NGT, silent generation, was performed via an extensive literature review with the aim of incorporating all peer-reviewed publications by the 13 institutions.

This review laid the foundation for stage two of the NGT, round robin, which was initiated by sending the literature review to all participating institutions prior to the in-person meeting. All institutions were asked to collect their ideas on the following five questions:

- What is the current top indication for open robotic-assisted microsurgery?
- What is the most beneficial feature of open robotic-assisted microsurgery?
- What is the top future indication for open robotic-assisted microsurgery?
- What should be the next improvement or add-on to the next generation of robots?
- What do you believe is the most valuable long-term goal for open robotic-assisted microsurgery?

Based on the collected answers, a hitlist of the four most common answers to the five questions was created. At the in-person consensus session at the EFSM 2024, the hitlists were presented to all participants. For each question, the round robin was completed by giving each individual center the opportunity to add to the hitlist.

During stage three, discussion, everyone had the opportunity to clarify the wording of the generated items and discuss the hitlist content.

In stage four, voting was divided into two stages. For the first sub-stage, each center received one vote to rank the four-item hitlist content with zero to three points with zero being the least important and three being the most important of the items. Each number from zero to three can only be given once within each question. The items scoring the highest for each question were then voted as the most important answer to the five questions with “agree” or “disagree.” If the rate of “agree” was > 70% of all participating centers, the item was called as consented.

## Results

### Preclinical training

Several studies have examined the learning curve of subjects with different levels of experience in microsurgery, ranging from complete novices to expert microsurgeons. van Mulken et al. introduced the MUSA robot in 2018 with promising learning curves of robot-assisted anastomoses on artificial vessels [12]. A successive rodent study confirmed patent robot-assisted micro-anastomoses in vivo with the MUSA robot [13] (Table 1). Ballestín et al. performed studies on artificial vessels with the Symani in 2022 and also confirmed a greater precision with longer anastomosis times with robotic assistance [14]. Two preclinical studies by Wessel et al. confirmed these findings as well as a learning curve assessed using the modified structural assessment of microsurgical skills (mSAMS) score [15, 16]. An in vivo preclinical study further demonstrated equal patency between manual and robotic anastomoses but showed decreased tissue damage by means of lower host reaction scores in histology [17]. Friedberg et al. evaluated the learning curve with the MUSA system in relation to the microsurgical experience of surgeons and concluded a steep learning curve among all participants. However, the robot helped novice and intermediate surgeons perform better technically with fewer errors then their hand-sewn anastomoses [18].

**Table 1:**
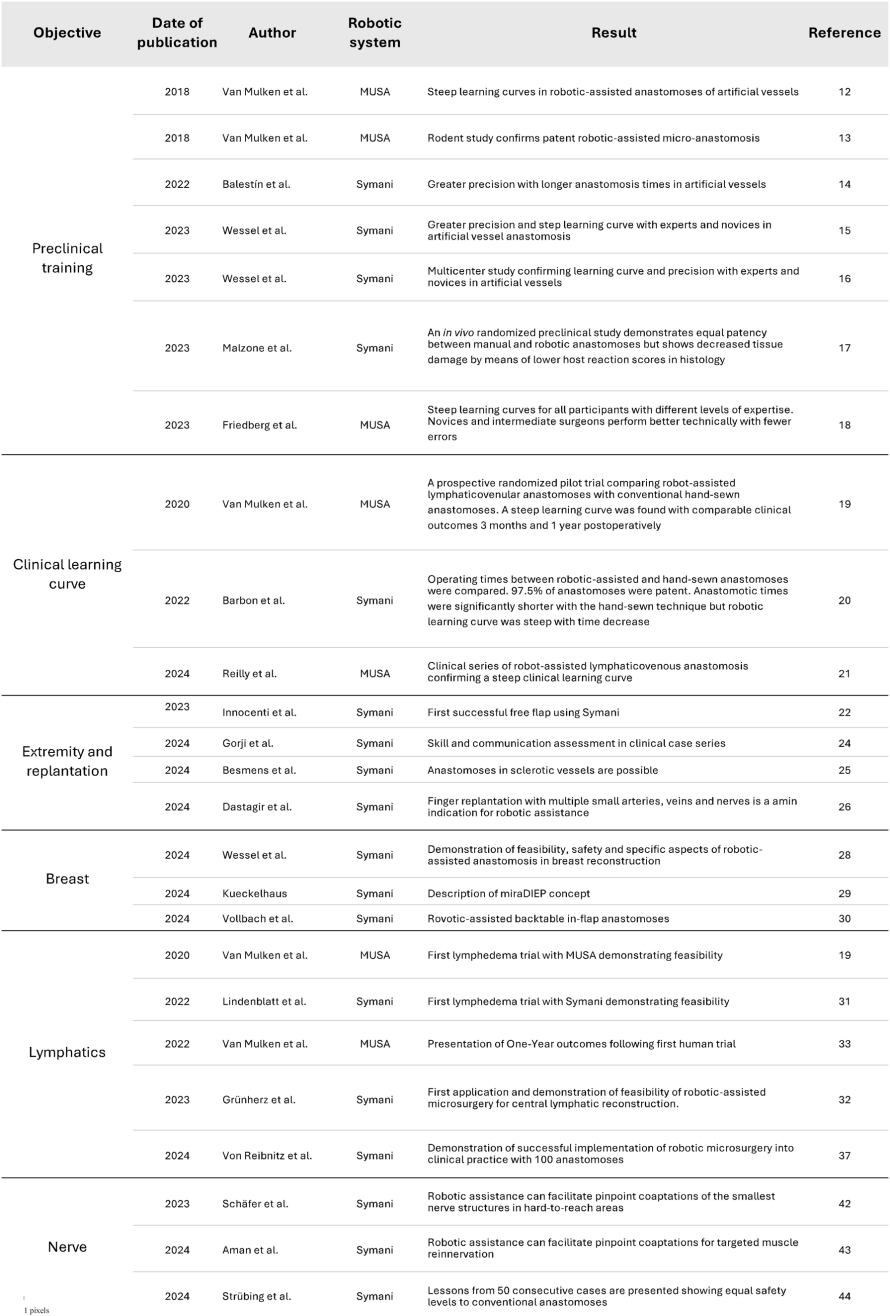
Key literature at a glance

### Clinical learning curve

The world’s first clinical trial of robot-assisted microsurgery was published in 2020 using the MUSA system, confirming the feasibility of robot-assisted supermicrosurgical anastomosis [19]. This prospective randomized pilot trial compared robot-assisted lymphaticovenular anastomoses with conventional hand-sewn anastomoses. Although the time to perform the anastomoses was longer in the robot-assisted group, a steep learning curve was found for both the time and the quality of the anastomoses with comparable clinical outcomes 3 months and 1 year postoperatively. Barbon et al. published the first results on the clinical learning curve using the Symani in 2022 [20]. Operating times between robotic-assisted and hand-sewn anastomoses (n = 32) were compared. 97.5% of anastomoses were patent. Anastomotic times were significantly shorter with the hand-sewn technique. However, the learning curve was steep, and the time needed to perform the anastomosis consistently decreased over time, to the point where, in the last operations, the times to perform lympho-venous anastomoses were comparable (8–10 min).

Reilly et al. published a clinical series of robot-assisted lymphaticovenous anastomosis using the MUSA system, also demonstrating a steep clinical learning curve. Moreover, they recorded decreased frustration and effort levels through a higher case volume [21].

### Extremity and replantation

The first free flap performed with Symani robotic assistance was reported by Innocenti et al. [22]. The emerging concept of perforator-to-perforator anastomosis in delicate free flaps [7, 23] demands for extensive microsurgical skills. Robot-assisted microsurgery has the potential to enable microsurgeons with limited clinical experience to perform such advanced techniques [24].

While thick or sclerosed vessel walls may present a specific challenge for robotic-assisted anastomosis in lower extremity reconstruction, this issue becomes less relevant as vessel size decreases. However, robotic-assisted anastomosis has still been shown to be feasible for sclerotic vessels [25].

**Fig. 1:**
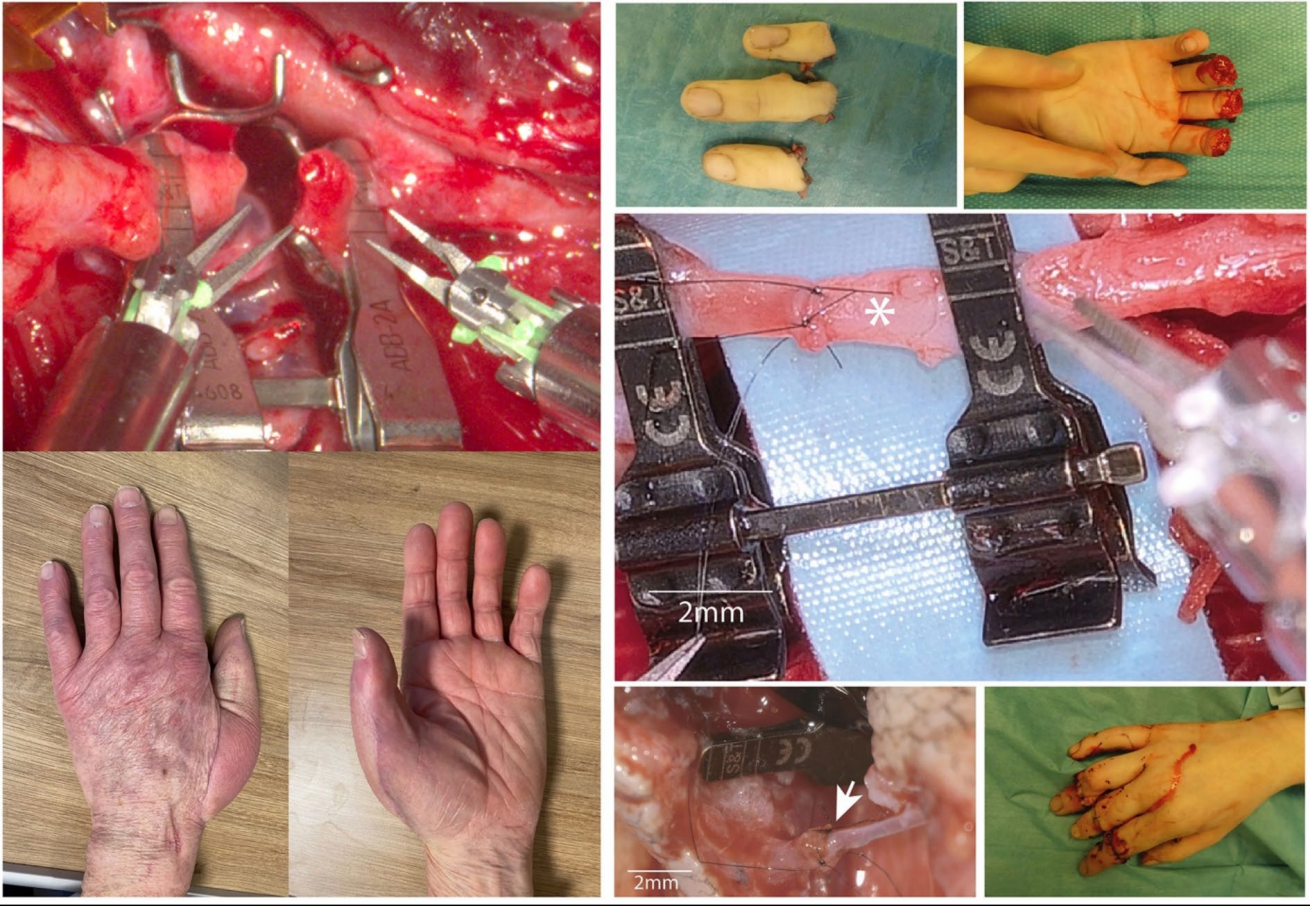
Upper extremity reconstruction: left top: robot-assisted SCIP flap for hand reconstruction with perforator-to-perforator anastomosis to a radial artery perforator with good size match; left bottom: robot-assisted SCIP flap to the dorsal metacarpal artery for thumb reconstruction; right: hand replantation involving 3 venous interpositions (6 anastomoses) and 3 end-to-end arterial anastomoses for arterial reconstruction. In addition, 9 veins were sutured, and 6 nerves were coapted with robotic assistance

Potentially optimal indications for robotic assistance are delicate free flaps for hand and finger coverage. Finger replantation with multiple small arteries, veins and nerves represents another main indication for robotic assistance (Fig. 1) [26]. Enhanced precision has the potential to improve outcomes in nerve repair and reconstruction [27].

### Breast

Feasibility and safety of robot-assisted breast reconstruction were demonstrated using Symani [28]. While performing robot-assisted anastomoses with either system, it was noted that thoracic excursions through ventilation resulted in the susception of a moving situs. This effect is due to the robot’s fixed position, while the hands for manual anastomoses usually rest on the moving thorax. These movements were more noticeable on the ventral thorax and could be reduced by tidal volume reduction. The predictable movements of machine ventilation allow surgeons to time microsurgical movements and limit the moving situs effect.

**Fig. 2:**
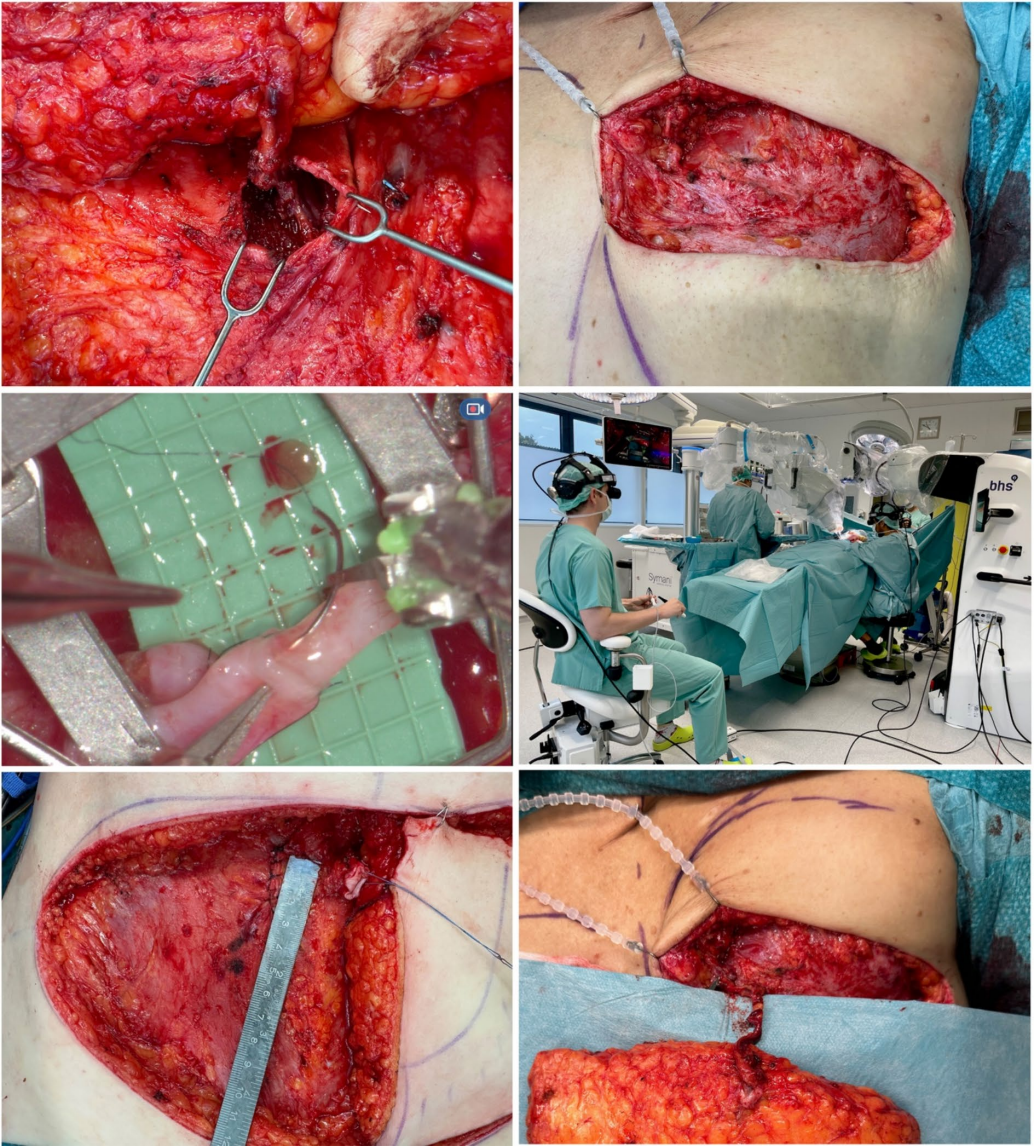
Breast reconstruction: minimally invasive robot-assisted DIEP flap breast reconstruction (miraDIEP) with perforator-to-perforator anastomosis. Top left: flap elevation via microfascial incision; top right: epipectoral IMAP for robot-assisted anastomosis; middle left: robotic instruments with distal motion axes during anastomosis of IMAP (right vessel) to DIEA (left vessel) with decent size match; middle right: surgeon (on the left) performs the anastomosis remotely while assistant is scrubbed in; bottom left: sutured facia following flap harvest; bottom right: flap after epipectoral anastomosis and reperfusion prior to inset

While robot-assisted DIEP flap elevation techniques using the da Vinci system were developed to minimize abdominal donor-site morbidity, another approach using open robot-assisted microsurgery is the miraDIEP (minimally invasive robotic-assisted DIEP) concept [29]. It combines a microfascial incision for a short DIEP pedicle harvest with a robot-assisted anastomosis to internal mammary artery perforators (IMAPs) to achieve minimal invasiveness (Fig. 2). The combination of a short pedicle with the IMAP created a decent size match, while the IMAP adds to the DIEP pedicle length.

Small in-flap anastomoses can also be comfortably performed with robotic assistance on a back table [30].

### Head & neck

Microsurgical vascular anastomosis is a common technique used to reconstruct lost facial regions using both bony and soft tissue transplants. Functional rehabilitation should aim to provide tailor-made solutions. Microsurgical nerve reconstructions are also important in the head and neck region, either as a simple reconstruction after traumatic or iatrogenic injury, or as nerve grafts for reconstruction after ablative tumor surgery. Microsurgery can be used to treat lymphedema and lymph fistulas after neck dissection. Robotic-assisted microsurgery could provide access to other indications, such as intraoral vascular anastomosis to facial and palatal vessels. Multicenter clinical experience with MUSA and Symani in the head and neck area remains to be published.

### Lymphatics

**Fig. 3:**
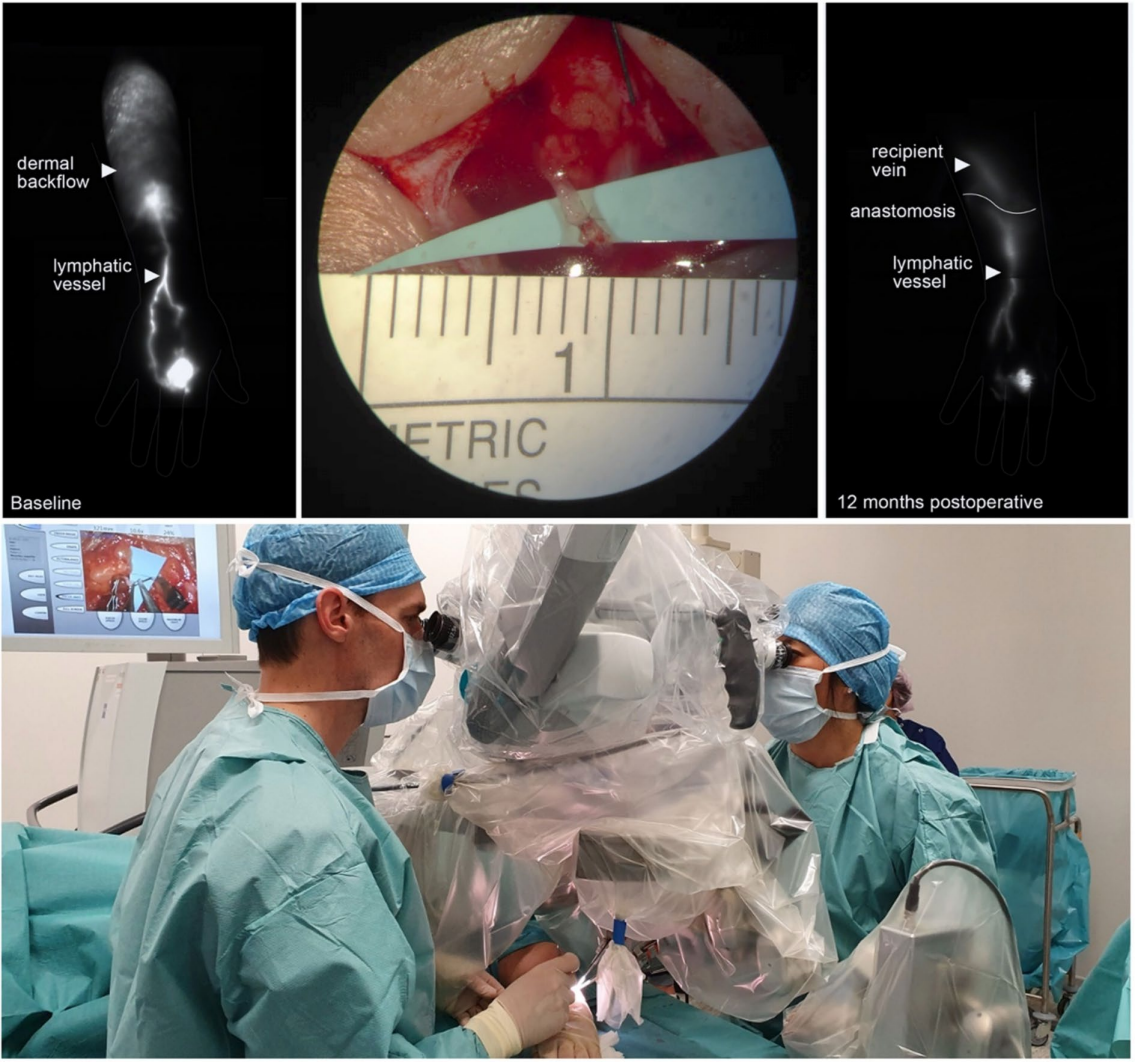
Robotic LVA under local anesthesia using the MUSA-2 system. Upper left: preoperative near-infrared fluorescence (NIRF) lymphangiography; upper middle: successful robotic-assisted lymphovenous anastomosis; upper right: NIRF lymphangiography demonstrating a patent anastomosis 12 months postoperatively; bottom: MUSA-2 during surgery with small footprint

**Fig. 4:**
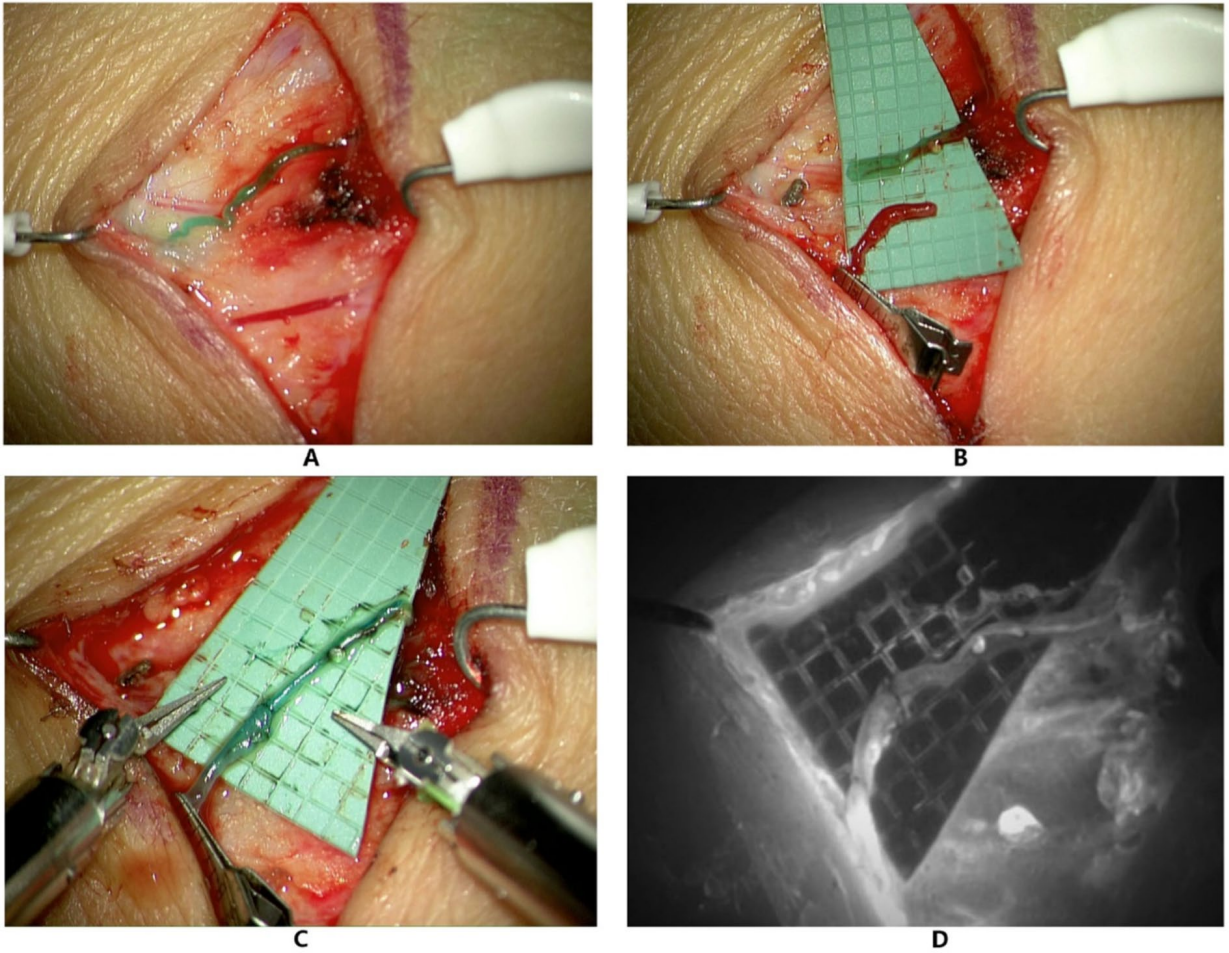
Presentation of a robotic-assisted lymphovenous anastomosis performed with the Symani Surgical System®. **A** 0.5 mm lymphatic vessel (above) and 0.5 mm vein (below) after intradermal injection of 0.2 mL Indocyanine green (ICG)/patent blue. **B** Proximal transection of the lymphatic and distal transection of the vein for end-to-end anastomosis. An intravascular stent (IVAS) was used for vessel stabilization during anastomosis. **C** Robot-assisted lymphovenous anastomosis with Nylon 11-0 showing good patency. **D** Fluorescent mode confirming lymphatic flow of ICG into the vein. Reused from35 (Fig. 1) under a creative commons license CC BY 4.0 https://creativecommons.org/licenses/by/4.0/

Robotic-assisted lymphatic micro- and super-microsurgeries currently represent the field that is most intensively studied and was the first area for which a dedicated robotic microsurgical system was designed. The first human trial using the MUSA robot was published in 2020 by van Mulken et al. in patients with breast cancer-related lymphedema [19]. Lindenblatt et al. published the first in-human study using the Symani for lymphatic reconstruction in early 2022 [31]. Subsequent follow-up studies showed a high feasibility of robotic microsurgical systems to perform lymphatic supermicrosurgery, showing distinct benefits when operating on very small vessels (< 0.8 mm) and in deeper anatomic planes and a steep learning curve [20, 21, 32–36]. von Reibnitz et al. reported the results of 100 consecutive anastomoses in 67 patients for lymphatic reconstruction, showing the feasibility of robotic-assisted anastomoses with low complication rates and good clinical outcomes [37]. On consideration of the delicacy of lymphatic vessels, robotic-assisted supermicrosurgery may be particularly beneficial to improve microsurgical technique due to its inherent motion scaling technique making reliable anastomoses on even smaller vessels than 0.3 mm more reliable in future (Figs. 3 and 4).

Long robotic arms operated remotely may also facilitate microsurgical reconstruction of the central lymphatic system, which lies deep within the body and have not been a part of common reconstructive procedures in the past. Recently, the first in-human use of the Symani has been reported for central lymphatic reconstruction [38]. In addition, the Symani has shown benefits in central lymphatic reconstruction at different anatomic levels [39]. Robot-assisted lymphaticovenular anastomosis can be performed safely under local anesthesia on an outpatient basis [21].

### Nerve

Meticulous nerve coaptation is crucial for successful reconstruction [40], especially with the advent of targeted muscle reinnervation (TMR) [41].

A recent study by Schäfer et al. demonstrated the feasibility of using Symani in peripheral nerve surgery concluding that Symani can facilitate pinpoint coaptations of the smallest nerve structures in hard-to-reach areas [42]. Another study performed multiple nerve transfers with the Symani in a patient suffering from traumatic transhumeral amputation, with a similar conclusion [43].

A prospective study by Strübing et al. analyzed 50 clinical cases [44] including four neurotized free flaps and another four TMRs after upper extremity trauma.

Transected digital nerve repair of the hand was performed with both the MUSA and Symani systems.

### Consensus results

As part of the result of the modified NGT’s stages I–III, the top four answers to five major questions were identified (Table 2).

**Table 2:**
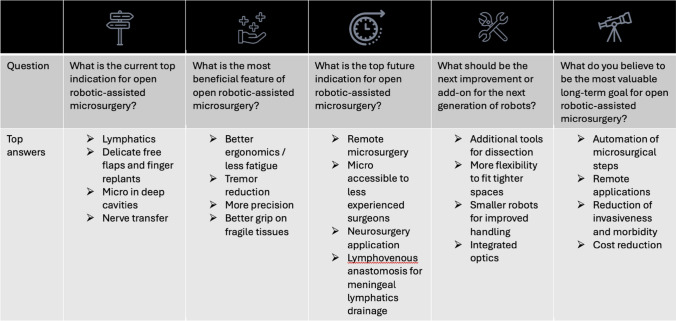
Five important questions regarding robotic-assisted microsurgery were asked following reviewing the literature published by all institutions actively performing robotic-assisted microsurgery using the MUSA-2 system or the Symani Surgical system

The four most common answers were displayed prior to consensus voting

In Stage IV, the answers were ranked in the first round of voting (Table 3). Then, the highest-ranked answer was voted again in voting round two to identify whether a consensus was reached. The top current indication for open robotic microsurgery was “lymphatic supermicrosurgery” receiving 49% of all voting points, followed by delicate free tissue transfer or replantation requiring small vessel anastomoses approaching the realm of supermicrosurgery with 33%, microsurgery in deep cavities with 12% and nerve transfer with 6% of all votes. One hundred percent of the participants agreed that “lymphatic supermicrosurgery” was the top current indication; therefore, a consensus was reached.

**Table 3:**
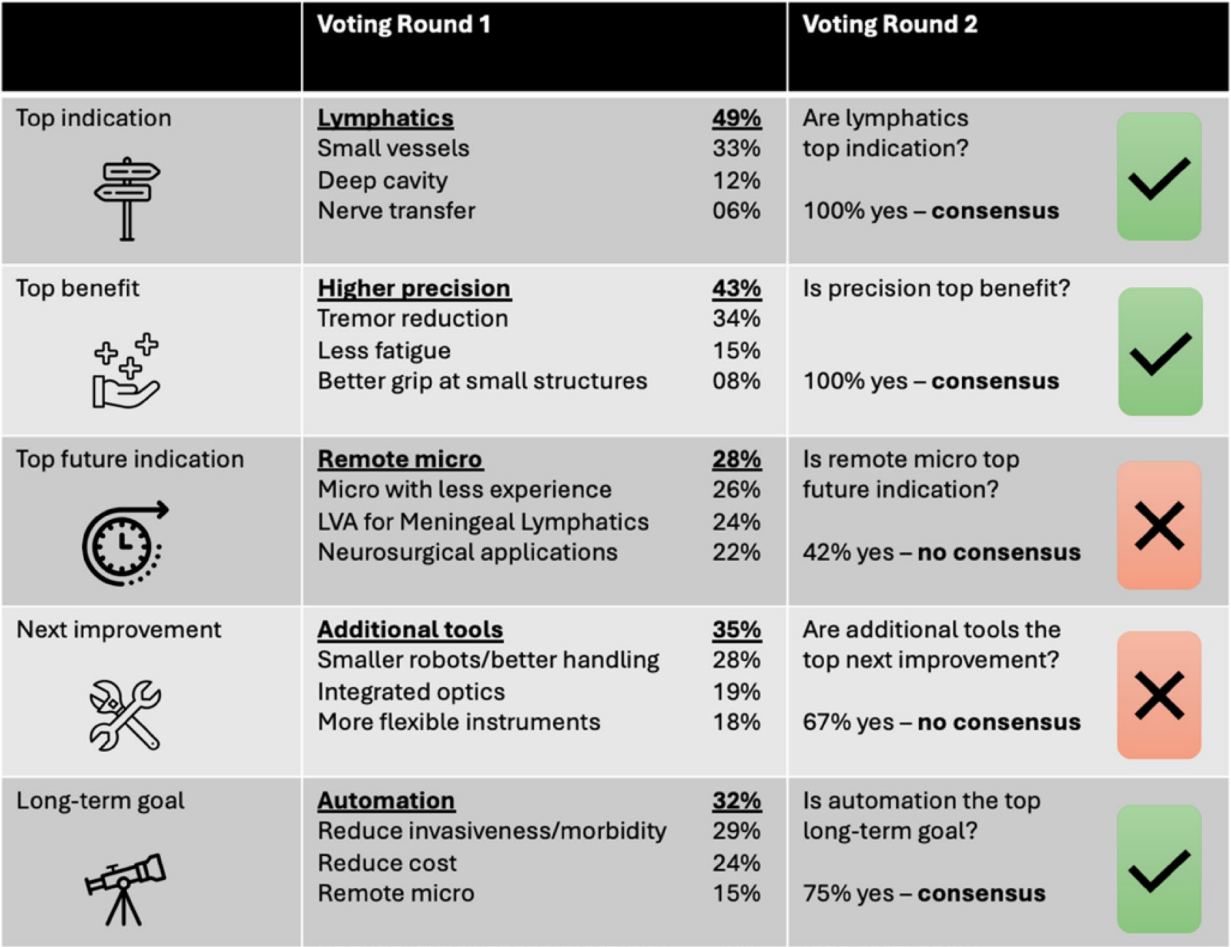
Two voting rounds were conducted in order to identify whether there is a consensus among the active centers or not. In round one, each center could allocate a defined number of points toward each of the four answers to each of the five questions

In voting round two, the answer with the most points was voted on again with the question if this is the best answer to the question. If over 70% of centers voted “yes” a consensus was achieved

As the top current benefit of robotic microsurgery, “higher precision” received 43% of all the voting points. Further, tremor reduction with 34%, less fatigue with 15%, and better grip for small structures completed the list. All participants agreed on “higher precision” being the top current benefit of robotic microsurgery; therefore, a consensus was reached.

In the voting top future indication, “remote microsurgery” received 28% of points, “enabling microsurgery for less-experienced surgeons” 26%, “lymphovenous anastomoses for drainage of meningeal lymphatics” 24%, and “neurosurgical applications” 22%. No consensus was reached regarding the top future indication.

In the vote for the most important next technological improvement, “additional tools” received 35% of voting points, “smaller robots/better handling” 28%, “integrated optics” 19%, and “more flexible instruments” 18%. No consensus was reached on what the most important next improvement was.

As for the most valuable long-term goal within the realm of robotic microsurgery, 32% of voting points went to “automation”. The reduction of invasiveness and morbidity received 29% while cost reduction scored 24%. Fifteen percent of votes went to “remote microsurgery”. In voting round two, 75% of the participants agreed that “automation” was the most valuable long-term goal for robotic microsurgery, so a consensus was reached.

## Discussion

Robotic-assisted open microsurgery is a clinical reality in Europe with a broad spectrum of applications and several centers performing these surgeries routinely. A comprehensive and consented clinical user guide for microsurgical centers implementing robotic assistance is provided.

### Differences between Symani and MUSA-2

Symani’s and MUSA-2’s specific configurations need to be considered when planning clinical procedures. While both systems provide tremor elimination and motion scaling, the Symani provides additional distal motion axes within the disposable instruments. This feature provides greater flexibility for needle and tissue handling in deeper planes [32, 38]. The advantage of MUSA-2 is that it can be combined with any (super-)microsurgery instruments that are connected into the system using small 3D-printed adapters, saving costs and waste of disposables [19]. In addition, the range of instruments that can be used in the MUSA robot is currently broader.

While the MUSA-2 controllers are connected mechanically, Symani has a telemetric control with free controllers being operated in an electromagnetic field.

Both systems are suitable for remote positioning of the surgeon if combined with a suitable optical system [45].

### Optics in open robotic-assisted microsurgery

Both robotic platforms can be combined with conventional microscopes [19, 22]. More flexible positioning can be achieved by combination with an (robotic) exoscope [15, 32, 33]. The operating field is visualized using 3D screens or via head-mounted displays [32, 46]. Several studies have suggested an ergonomic benefit of this combination with such optical systems [15, 47].

### Learning curve

Preclinical trials have confirmed a step learning curve [14, 15, 17] and retrospective clinical studies have confirmed the presence of a subsequent clinical learning curve [19, 20].

Due to the lack of haptic feedback when using robotic assistance, surgeons need to develop a compensatory “see-feel” [20, 31].

### Indications and new techniques

Several new techniques have been developed since the European approval for clinical application has been obtained. The Symani’s unique capability to perform highly precise and complex motions in deep spaces led to the description of robotic-assisted central lymphatic reconstruction, making use of these exact features [38],^38^. Considering high morbidity and rarity of pathologies of the central lymphatic system, central lymphatic surgery has been rarely performed. Robotic-assisted microsurgery has great potential to expand the treatment options for central lymphatic lesions. Another technique utilizing the robotic precision is the miraDIEP concept, enabling minimal invasiveness for autologous DIEP flap reconstruction through robotic-assisted perforator-to-perforator anastomosis [48]. Generally, the smaller the structures for anastomosis/coaptation, the more prominent are the benefits of robotic assistance. This was confirmed by expert voting with lymphatics as the top indication and the provided precision as the top benefit of robotic-assisted microsurgery. In addition, following the description of the brain`s lymphatic system and its potential role in the development of neurodegenerative disease, robotic-assisted micro- and super-microsurgeries may facilitate lymphatic reconstruction of even smaller lymphatics in extra or even intracranial locations [49, 50].

### Clinical trials

Currently conducted prospective clinical trials using the MUSA system include an extension of the prospective randomized controlled pilot study comparing robot-assisted versus conventional lymphovenous anastomosis for breast cancer-related lymphedema. In addition, prospective clinical trials are being performed on digital nerve repair, free-flap extremity and breast reconstruction and digital replantation.

Prospective clinical trials with the Symani system include a randomized controlled pilot study comparing robot-assisted versus conventional lymphovenous anastomosis for breast cancer-related lymphedema [34]. Another trial currently recruiting is the PRIMO Post-Market Clinical Follow-Up Study, which is an open-label, single-group interventional study monitoring the safety and performance of Symani in free flaps, replantation, and lymphatic reconstruction (NCT04843436) [51].

### Current limitations and potential future improvements

The initial users encountered several aspects that need to be taken into consideration:

#### Grip strength

Grip strength noted could be stronger for larger vessels exceeding 2 mm in diameter as needle twisting can occur during stitching in both the Symani and the MUSA-2 systems. However, when operated in its intended fashion, needle twisting can be prevented by avoiding tension on the needle. In the MUSA system, grip strength limitations were only found in sclerotic vessels in lower extremity free-flap reconstructions. This could be solved by making use of the possibility to use any conventional microsurgical tool with the MUSA system. In general, the smaller the vessel and needle, the higher the relative grip strength.

#### Speed

Symani recognizes unintended movements and stops tracking as a safety mechanism. This feature works very reliably, but also limits the allowed speed of intentional movements. The MUSA system provides the ability to switch between the slow and precise scaled motion and the faster motion during the operation. The system uses two foot pedals, one accommodating a slow scaling factor and the other with no scaling factor.

#### Electromagnetic field

The electromagnetic field tracking Symani’s controllers is sufficiently large for most movements. However, an even larger field may decrease times for thread pulling and the number of decoupling events required for hand repositioning during the procedure. The master manipulators of the MUSA system do not use electromagnetic fields.

#### Motion scaling

The available motion scaling factors of both robotic systems are sufficient for all current indications. However, this may change in future with the introduction of new indications.

#### Fixed position

As the robot is in a fixed position during surgery, patients’ body movements become more apparent than in conventional microsurgery. This needs to be considered and compensated for especially in breast reconstruction. In addition, due to the fixed angle in which both arms of the system are related to each other, it may be difficult to reach certain areas deeper within the body.

### Future technology, indications and outlook

Future indications could be the harvest of delicate flaps at the suprafascial level and pedicle dissection. This requires the integration of electrocautery capabilities for dissection, coagulation and cutting, and certain grasping capabilities. Another indication could be recipient-site perforator preparation as well as preparation of lymphatics and veins. Such tools received most voting points in the consensus voting indicating their high relevance.

System size reduction will lead to better handling and hold promise for more flexibility and reduction of surgery times. Integrated optics could also lead to a more simplified setup. Additional flexibility in robotic instruments will be added to their application in even deeper and smaller spaces.

Future robot generations could enable customization for each specific user in scaling factors, speed limits, and grip strength and provide defined default settings for specific indications.

A depth recognition and compensation system could smooth out thoracic excursions.

Generally, robotic assistance in microsurgery holds promise to open this field to a wider audience of surgeons, potentially providing a steeper learning curve for complex supermicrosurgical procedures. The neurosurgical field may benefit the surgeons once the approval for the application of such robotics is obtained.

Remote operations may enable experts in specific steps of a microsurgical procedure to apply their expertise without having to be on site. An automation of specific surgical steps, potentially supported by machine learning and artificial intelligence, holds promise for simplifying complex procedures. Although there are many technical and regulatory hurdles to overcome, these long-term goals may enhance surgical outcomes. Reducing invasiveness and morbidity may ultimately lead to cost reduction despite the added cost of the necessary technology. Cost–benefit analysis should be integrated in future studies to evaluate clinical benefits, help guide healthcare stakeholder decision-making, and drive down technology costs.

## Supplementary Information

Below is the link to the electronic supplementary material.

Supplementary file1 (DOCX 5791 KB)

## Data Availability

No datasets were generated or analysed during the current study.
