# Supplementary material for: Clinical experience in open robotic-assisted microsurgery: user consensus of the European Federation of Societies for Microsurgery
Source: J Robot Surg. 2025 Apr 22;19(1):171. doi: 10.1007/s11701-025-02338-w (PMC12014844; doi:10.1007/s11701-025-02338-w)

Supplemental digital content


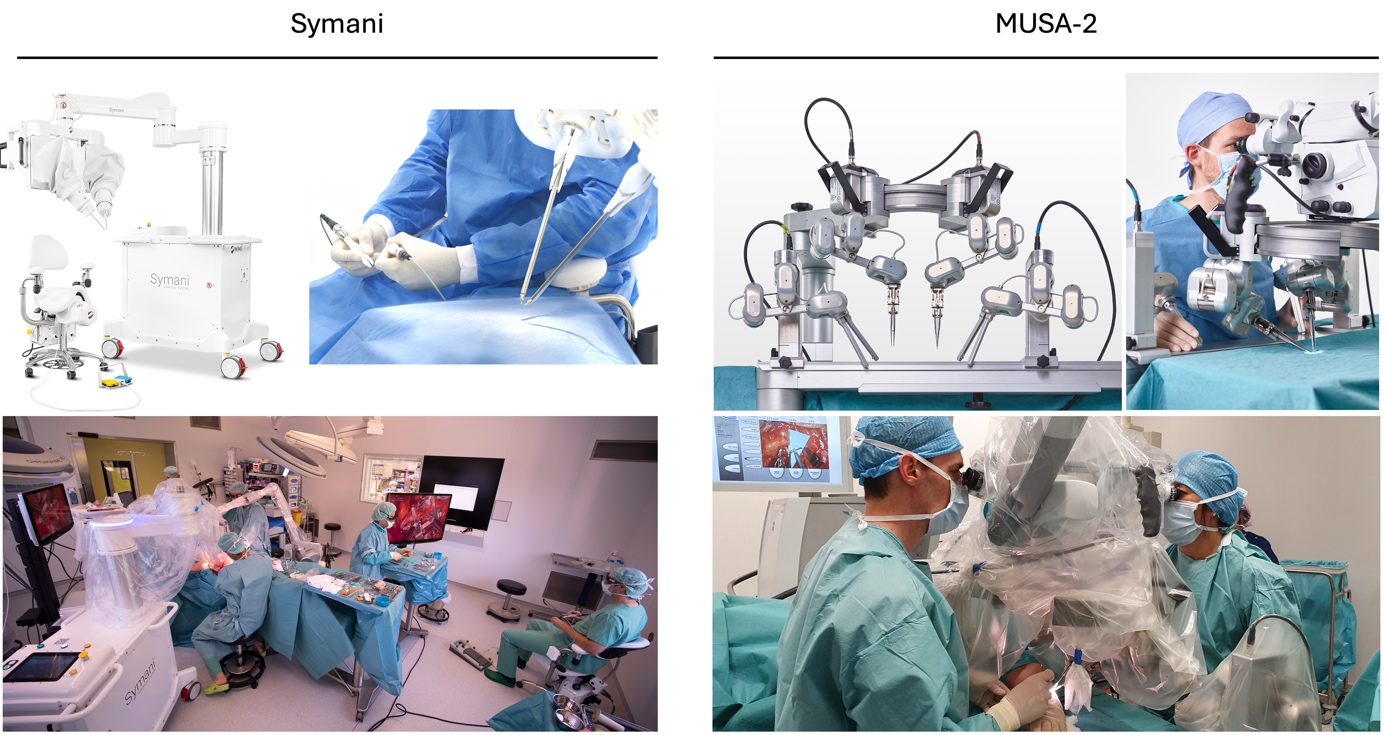


Suppl. Figure 1: upper panel: Symani and MUSA systems (images provided by MMI and Microsure); lower panel: Systems setup during surgery


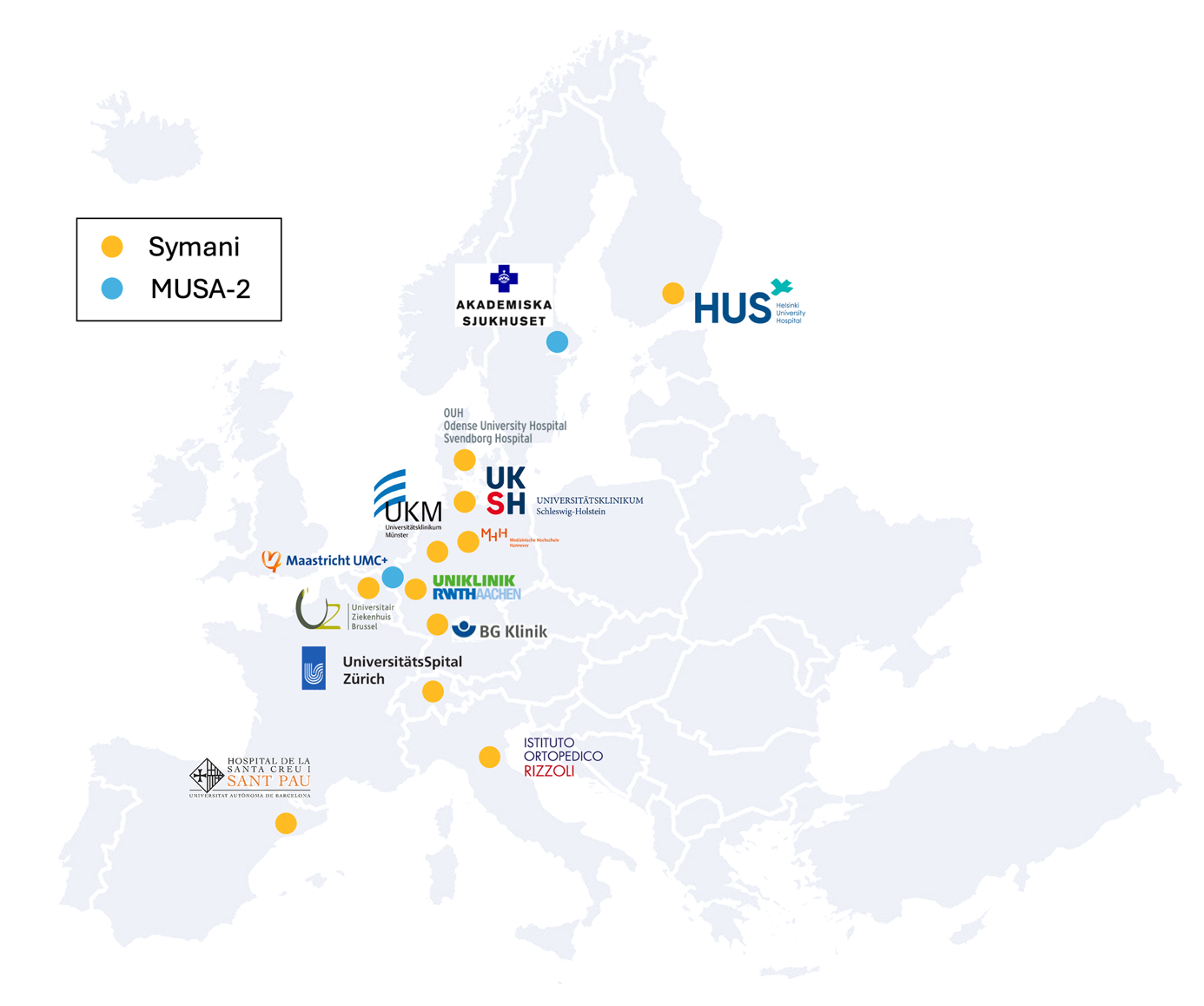


Suppl. Figure 2: All centers performing surgery using the Symani or the MUSA-2 robots (by April 1^st^ 2024) on map of Europe


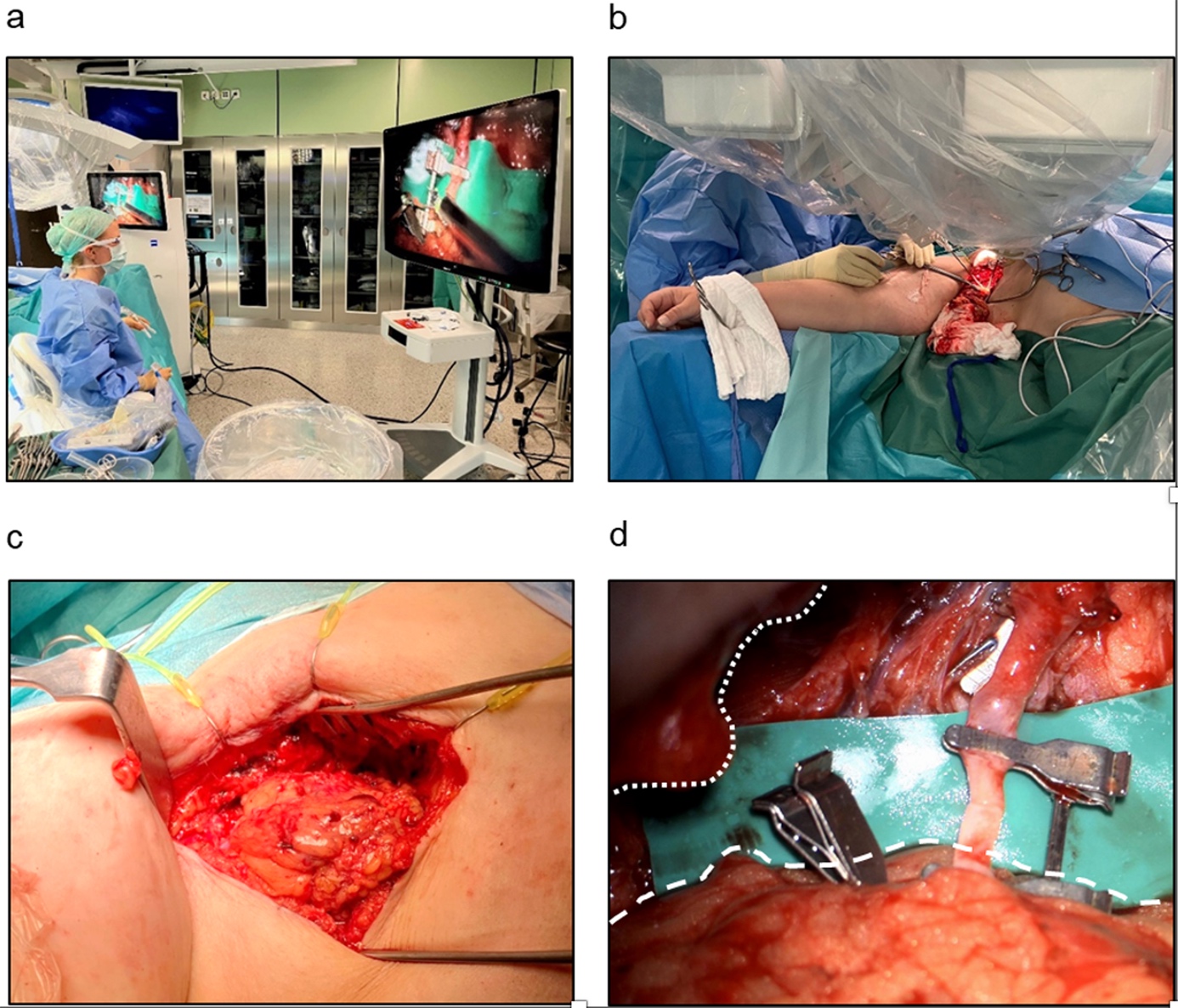


Suppl. Figure 3 (a) Operative setup in which the surgeon operates the Symani® Surgical System using handheld manipulators and the KINEVO® 900 exoscope using 3D visualization. (b) An assistant can be seen next to the operating field while the robotic arms are in place. (c) Access to the recipient vessel through the axilla with the omental flap. (d) The obstructed view by the entry to the cavity of the axilla (dotted line) and the voluminous tissue transplant (broken line) that is well managed by robotic-assisted surgery is notable Reused from^32^ (Figure 2) under a creative commons license .CC BY 4.0 <https://creativecommons.org/licenses/by/4.0/>

Suppl. Table 1: Center participating in the consensus session at EFSM 2024 in Milano (red: Symani Surgical System; blue: MUSA-2 system).


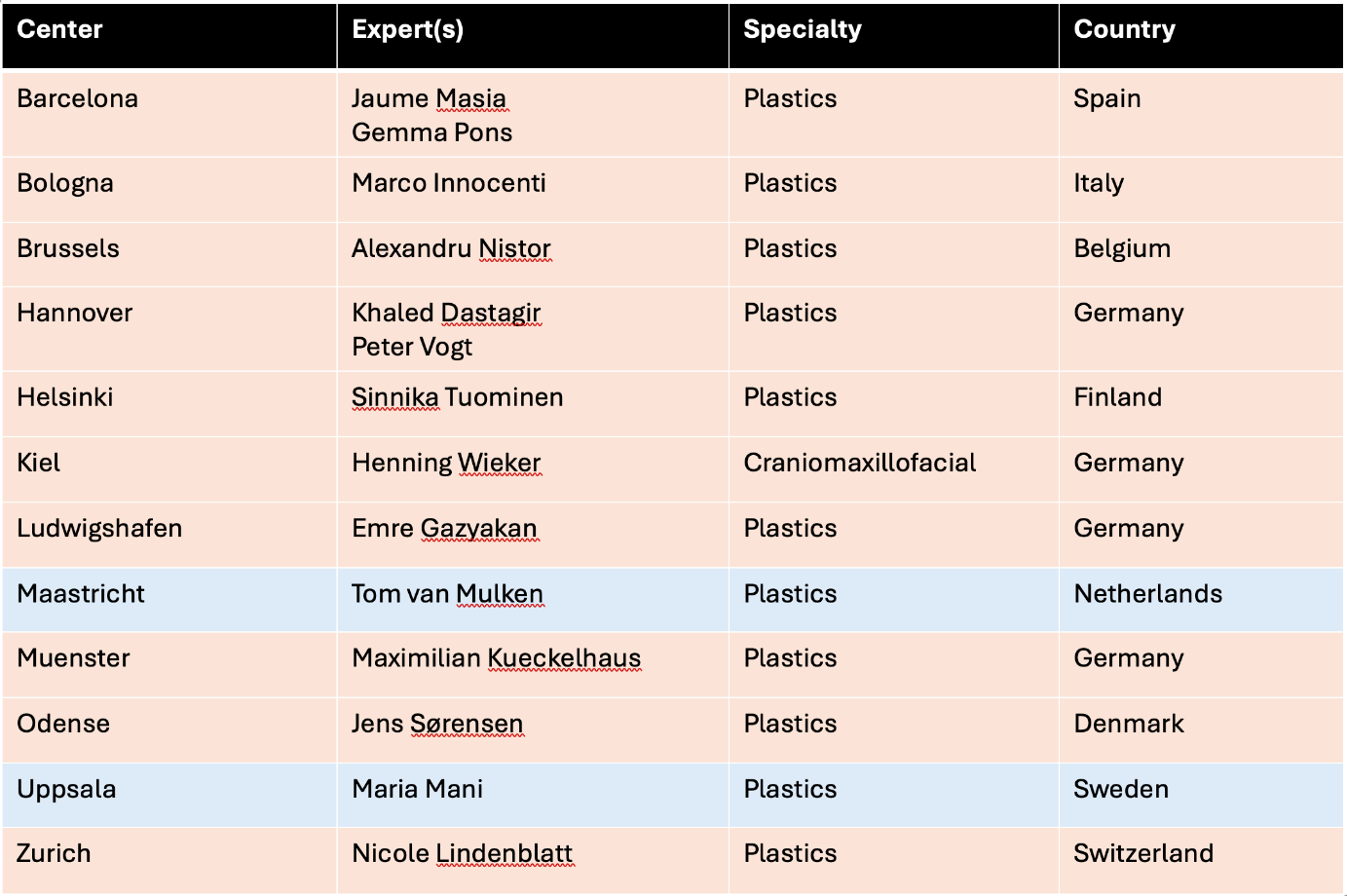

Supplement: Supplementary file 1 — Supplementary file1 (DOCX 5791 KB) [file 11701_2025_2338_MOESM1_ESM.docx]
